# Mitochondrial and Antioxidant Activity as the Basis of the Neuroprotective Effect of Potential Multitarget Drugs for the Treatment of Neurodegenerative Diseases

**DOI:** 10.2174/011570159X409525251027064739

**Published:** 2026-01-19

**Authors:** Elena Feofanovna Shevtsova, Alexey Yuryevich Aksinenko, Alla Vadimovna Stavrovskaya, Vladimir Petrovich Fisenko, Sergey Olegovich Bachurin

**Affiliations:** 1 Institute of Physiologically Active Compounds at Federal Research Center of Problems of Chemical Physics and Medicinal Chemistry, Russian Academy of Sciences (IPAC RAS), Chernogolovka, 142432, Russia;; 2 Research Center of Neurology, Ministry of Science and Higher Education of the Russian Federation, Moscow, 125367, Russian Federation;; 3 Department of Pharmacology, I.M. Sechenov First Moscow State Medical University, 8 Trubetskaya St., 119991, Moscow, Russian Federation

**Keywords:** Neurodegenerative diseases, mitochondria, mitochondrial permeability transition, antioxidant activity, neuroprotectors, multitarget drugs

## Abstract

**Background:**

The mechanisms of neurodegeneration common to many neurodegenerative diseases include oxidative stress, mitochondrial dysfunctions, excitotoxicity, and others. Beyond the broad spectrum of strategies for developing neuroprotective agents that target stage-specific mechanisms in each neurodegenerative disease, considerable attention is also being given to approaches aimed at developing compounds that can effectively modulate general pathogenic mechanisms and enhance the overall resilience of neuronal cells to cell death induction.

**Objective:**

This review discusses some of the results on new multitarget multipharmacophore agents with neuroprotective effects, particularly through their influence on mitochondrial permeability transition and antioxidant properties. We conducted comprehensive online searches on PubMed to gather the latest data on multitarget multipharmacophore agents consisting of pre-defined pharmacophores that have already demonstrated neuroprotective properties.

**Results and Discussion:**

To create compounds with a desirable spectrum of biological activity, an approach based on the conjugation of specific structural fragments of pharmacologically active substances into a single molecular entity could be used. Core fragments of compounds that have already demonstrated neuroprotective properties due to mitochondrial and antioxidant mechanisms of action can be used as neuroactive scaffolds.

**Conclusion:**

The combination of several pharmacophores in one molecule may not only result in the mere addition of the useful properties of each component, but may also give rise to new types of biological activity. The examples of the appearance of new properties in such multipharmacophore compounds, not inherent in the reference agents, discussed in our review, may be considered a prospective approach for creating a novel generation of neuroprotective agents.

## 1 INTRODUCTION

Neurodegenerative diseases (ND) represent a broad group of disorders caused by invariably progressive, devastatingly debilitating neuronal cell death. Of particular importance among different ND are Alzheimer’s disease (AD), Parkinson’s disease (PD), and amyotrophic lateral sclerosis (ALS), as the most common and ultimately lethal [1]. A significant amount of funds and effort is spent every year on the development and clinical trials of new potential drugs for the treatment of these NDs [2]. Nevertheless, only a few medicines are used in real therapeutic practice to date including four low molecular weight synthetic drugs (donepezil, rivastigmine, galantamine and memantine) and 3 antibody-based therapeutics for AD treatment [3], 3 agents for ALS (riluzole, edaravone, and tofersen) [4], and some more agents for PD based mainly on different combinations with levodopa [5]. In all cases, the therapeutic effect of these agents is very limited, as they work only at the symptomatic level.

In recent years, special attention has been paid to the discovery of a new generation of agents and approaches that modulate key steps specific to the pathogenesis of ND and therefore could slow down or even prevent the development of the entire neurodegenerative process [3, 6].

The alternative approach to preventing ND development involves developing compounds that can efficiently modulate common mechanisms in the cascade of pathogenic events in ND and enhance the general resilience of neuronal cells against cell death. Among these general neurotoxic processes are glutamate-induced neuronal death (excitotoxicity) [7, 8], oxidative damage, reduced antioxidant potential of neuronal cells [9], and decreased mitochondrial resistance to inducers of the mitochondrial permeability transition (MPT) process [10]. The latter two mechanisms are fundamental in the development of age-related forms of neurodegenerative diseases [11-13].

Mitochondria and the MPT pores opening, which play a crucial role and may be the point of no return in the cell death cascade, are of great interest as targets [10, 14].

 The effect of “mitoprotection” can be achieved by different ways: either as a result of direct inhibition of MPT pore formation, or as a result of a decrease in calcium uptake by mitochondria, which in turn can occur due to the inhibition of specific calcium transporters, or due to a decrease in mitochondrial potential as a result of mild uncoupling [14-16].

In addition, neuroprotection can be provided by the prevention of lipid peroxidation that increases the resistance of mitochondria to the induction of the МРТ [12]. The phenomenon of non-specific permeability of mitochondrial membranes occurs due to the formation of a multiprotein complex and is closely associated with the initiation of cell death [17]. The composition of the pore has been a matter of active discussion for many years, and more details can be found in the reviews [18-21]. Among the components and direct regulators of the pores suggested by various researchers are: the F0 subunit of ATP synthase, an adenine nucleotide transporter, VDAC, several other mitochondrial transporters, BCL family proteins, creatine kinase, TSPO protein, and, on the other hand, a group of amyloidogenic proteins. [18, 22, 23]. The only undoubted non-pore-forming component and regulator of MPT pores is cyclophilin D (peptidyl-prolyl cis-trans isomerase), the ligand of which is cyclosporin A, a specific inhibitor and marker of MPT [24, 25]. A necessary inducer of MPT pore opening is calcium, or more specifically, the exceeding of a threshold by the intramitochondrial concentration of free calcium [26]. Threshold calcium concentrations can be reduced under conditions of oxidative stress, accompanied by accumulation of oxidative damage to mitochondrial membranes, increased free phosphate levels, and various impairments of the mitochondrial respiratory chain [27, 28]. On the other hand, for excitable brain cells, the calcium-buffering function of mitochondria is critical, and temporary low-amplitude MPT pore opening plays a crucial role in conducting neuronal stimuli, adapting metabolic pathways to the calcium-dependent activity of neuronal cells, and forming neuronal plasticity [29-32]. A decrease in the potential-dependent calcium entry into mitochondria by blocking calcium transporters, particularly MCU, or by effects leading to mitochondrial depolarization, such as uncoupling of oxidative phosphorylation, may also be a mechanism for inhibiting the opening of MPT pores.

We conducted comprehensive online searches on PubMed to gather the latest data on multitarget multipharmacophore agents consisting of pre-defined pharmacophores that have already demonstrated neuroprotective properties.

This review discusses selected results from the authors and other laboratories on the design and study of original conjugated compounds that exhibit mitoprotective and antioxidant properties, making them potential neuroprotective agents. To create compounds with a desirable spectrum of biological activity, an approach based on the conjugation of specific structural fragments (“pharmacophores”) of pharmacologically active substances into a single molecular entity could be used. Such chimeric agents could be referred to as “multipharmacophore” compounds [33, 34]. Core fragments of compounds that have already demonstrated neuroprotective properties in various *in vitro* and *in vivo* models can be used as neuroactive scaffolds (Fig. **1**), in particular: the phenothiazine fragment of the agent Methylene Blue (MB), which has recently been considered a potential candidate for neuroprotective drugs [35-40]; the aminoadamantane fragment of the well-known anti-AD drug memantine and the antiparkinsonian agent amantadine, both acting as voltage-dependent low-affinity ligands for NMDA glutamate receptors [41-44]; the carbazole fragment, which represents the core structure of several recently identified agents with pro-neurogenic and mitoprotective effects [45-47]; and the γ-carboline fragment of the agent Dimebon, which has shown the ability to prevent the progression of neurodegenerative processes in various *in vitro* and *in vivo* models of AD, ALS, and other proteinopathies [48-50].

 Various synthetic algorithms can be used to synthesize original conjugated structures. Some of them are schematically shown in Fig. (**2**). The first – “acrylate” method of synthesis includes preliminary preparation of acrylate-containing pharmacophores and the subsequent Michael alkylation of pharmacophores containing an amino group. The second – “epoxide” method implies the use of epoxide-containing pharmacophores as precursors and subsequent alkylation with pharmacophores containing an amino group. The third – “mesylate”- method is based on the synthesis of an intermediate alkyl mesylate pharmacophore, in which the mesyl group is replaced *via* amination with another pharmacophore [51-56].

## 2 CONJUGATES OF TETRAHYDRO-ɣ-CARBOLINE AND PHENOTHIAZINE DERIVATIVES

Phenothiazine (10*H*-dibenzo-[b, e]-1,4-thiazine) derivatives, particularly Methylene Blue (MB), which was developed as an antimalarial agent, have been studied for many years as promising agents for protection against oxidative stress, ferroptosis, protein aggregation, and related disorders [57-59]. In various *in vitro*, *ex vivo*, and *in vivo* models, it has been demonstrated that MB has potential for treating pathologies such as Alzheimer’s and Parkinson’s diseases, as well as other neurodegenerative conditions [60-64].

In 2009, the strong neuroprotective effect of a combination of methylene blue (MB) with the antihistamine agent Dimebon - a tetrahydro-γ-carboline (2,3,4,9-tetrahydro-1H-pyrido [3]indole) derivative was demonstrated in a cell culture model of amyotrophic lateral sclerosis (ALS) [40]. It was revealed that Dimebon and MB can significantly reduce the levels of pathological TDP-43 aggregates in SH-SY5Y cells expressing TDP-43. Moreover, the joint application of both compounds reduced protein aggregation to a greater extent than would be expected from the mere summation of their effects.

As a further development of this research, an attempt was made to create new, original molecules that would combine the phenothiazine core of MB with the tetrahydro-γ-carboline moiety of Dimebon in a single molecular structure. Synthesis of conjugates of phenothiazines with γ-carboline or carbazole derivatives was described earlier [54, 65, 66]. The main chemotypes of the newly synthesized compounds are shown in Fig. (**3**).

Most conjugates containing the MB fragment showed high antioxidant properties, like МB itself, in contrast to conjugates with an unsubstituted phenothiazine ring.

Lead-compound – I**a** (Fig. **4**) in series **a** (Fig. **3**) demonstrated antiradical activity close to or exceeding that of Trolox in the ABTS assay and scavenged the peroxyl radical 7–10 times more effectively than Trolox in the ORAC assay [65, 67]. The antioxidant potential of this conjugate was also confirmed by inhibition of the Fe^3+^-induced LP in mitochondria, with IC_50_ being in the range of 0.7-3.2 µM, which is comparable with the activity of leuco-MB. It can be concluded that the electron-acceptor properties of МB account for its conjugate's ability to bypass the action of an inhibitor of the mitochondrial respiratory chain.

The prevailing contribution of MB to the activity was also shown for the leader compounds from a series of its conjugates with cycloalkaneindole derivatives (series **c-f**), in particular, one of the leader compounds – the MB conjugate with trifluoromethyl-containing tetrahydrocarbazole (compound **Ib**, Fig. **4**) [68]. In addition to antioxidant activity, this compound, like MB, prevents calcium-induced mitochondrial swelling, suggesting inhibition of the mitochondrial permeability transition. Furthermore, even in nanomolar concentrations, it exhibits neuroprotective properties in a cellular model of calcium stress on cortical primary neurons in the presence of ionomycin, a calcium ionophore [67]. Generally, compounds of this series are fairly close to or slightly exceed MB in the antioxidant properties and have pronounced mitoprotective activity. In particular, prevent mitochondrial membrane lipid peroxidation, restore electron transport chain impairment with the complex 1 inhibitor rotenone, and reduce calcium-induced MPT. As a result, they exhibit an obvious mitoprotective effect that also provides neuroprotective benefits in a cellular model of calcium overload using ionomycin [68]. Moreover, the shift away from a six-membered ring in the tetrahydrocarbazole fragment results in a significant reduction in both antioxidant and mitoprotective properties.

It is important that some of the studied conjugates, in particular compound **Ib** (Fig. **4**), in addition to the above-mentioned antioxidant and mitoprotective properties, also demonstrated the ability to stimulate the formation of straight, long microtubules with a normal structure, thereby compensating for degeneration resulting from taupathy [68].

The ability of some lead compounds in a series of γ-carboline and phenothiazine conjugates to affect pathological protein aggregation was confirmed in a cell model of proteinopathy, namely, cells overexpressing the FUS protein, which is a cellular model of one familiar form of ALS [69-71]. Fig. (**5**) shows micrographs of SH-SY5Y human neuroblastoma cell culture transfected with green fluorescent protein (GFP)-tagged FUS. Protein aggregates formed in the control culture are denser than the aggregates formed in the cells treated with Dimebon, МB, or compound **Ia** (Fig. **4**), which induce dispersion of dense aggregated structures (Fig. **5**).

Altogether, it can be concluded that structures based on phenothiazine conjugates with tetrahydro-gamma-carbolines and carbazoles could be discussed as a potential platform for developing new, promising neuroprotective agents that can block both disease-specific stage of ND (*e.g*. FUS protein aggregation at ALS) and such general stages in the ND pathogenesis as oxidative stress and mitochondrial dysfunction – namely vulnerability to MPT (Table **1**).

## 3 CONJUGATES OF TETRAHYDRO-Ɣ-CARBOLINE AND AMINOADAMANTANE DERIVATIVES

Another group of promising multipharmacophore agents represents conjugates of aminoadamantanes (tricyclo[3.3.1.13, 7]decan-2-amine) - the key pharmacophore of memantine and amantadine, widely used drugs for the treatment of Alzheimer's and Parkinson's diseases, respectively [5], and tetrahydro-γ-carbolines (THC) - the core pharmacophore fragment of the neuroprotective agent Dimebon [48].

γ-Carboline alkaloids are a family of natural and synthetic agents that exhibit biological activity *via* diverse mechanisms, particularly due to their mitoprotective, antioxidant, and anti-NMDA activities [73]. The most well-known representative of this group of compounds is Dimebon (Latrepirdine), which has neuroprotective properties and the ability to inhibit MPT, and has been widely studied [49, 74-78]. It was also demonstrated that bivalent gamma-carbolines exhibit neuroprotective activity in a cellular model of calcium overload, resulting from the suppression of MPT pore opening [79].

In another study [80], an N-methylated lead compound from the series of homobivalent gamma-carbolines was selected, which prevents excitotoxic cell death and inhibits calcium currents through NR1-1a/NR2A glutamate receptors. The severity of these effects is closely related to the presence of N-methylated pyridine in the structure. It should be noted that the IC_50_ of the compound-leader about glutamate receptors is 25 times higher than the IC_50_ concerning glutamate-induced cell death. This allows us to assume that, in this case, an additional mechanism of neuroprotection may be the effect on the MPT.

The second pharmacophore, the aminoadamantane fragment, is the key core of memantine and amantadine, whose neuroprotective effects are primarily linked to their ability to block the intracellular site of the NMDA receptor and thereby prevent the development of excitotoxic events in neurons. It was also shown that memantine inhibits calcium-induced MPT and increases mitochondrial calcium retention capacity [16], induces mitophagy and autophagy, and prevents disturbances in mitochondrial dynamics in the presence of mitochondrial toxins [81].

Earlier, it was shown that the agent amantadine, under conditions of impaired mitochondrial function due to HCV infection, can restore mitochondrial potential and increase calcium threshold required to trigger mitochondrial permeability transition opening, thereby reverting the HCV protein-mediated decrease in mitochondrial oxygen consumption and complex I and V activity [82]. This compound must not affect mitochondrial functions in control cells. Some experimental data on the similarity in the recovery of mitochondrial potential induced by amantadine and ruthenium red suggest that amantadine can inhibit the mitochondrial calcium uniporter (MCU) [82].

Earlier, we have described a quite broad spectrum of a new group of (γ-carboline and aminoadamantane derivatives (Fig. **6**) [83]. As expected from the presence of an aminoadamantane fragment, the compounds show notable activity as blockers of the NMDA-receptor intrachannel site. Additionally, some of them manifest significant, unexpected activity as uncompetitive blockers of the peripheral (ifenprodil-binding) site of this receptor.

Synthesis of this group of conjugates is described in a study [84].

For the compounds that contain a dimethylaminoadamantane fragment, the affinity to the MK-801-binding site was similar to that of memantine, for which this binding site is the major target. It was shown that the structure of the spacer also plays an important role in the activity of these compounds as NMDA receptor ligands: ethylene-bridged compounds **IIa**, unlike 1-oxoethylene-bridged compounds **IIb** (Fig. **6**), had similar micromolar IC_50_ values irrespective of the nature of the substituents in the conjugated scaffolds. It is noteworthy that the conjugates acquire the ability to bind to the ifenprodil site of the NMDA receptor, in contrast to memantine and Dimebon. The lead compounds (**IIa**: R = H; R1 = CH_3_) showed affinity toward both NMDA binding sites: Ki = 1.9 μM and 1.7 μM at the MK-801 and ifenprodil binding sites, respectively [84]. Thus, in this case, the design of conjugated structures gives rise to new properties that are absent in the compounds containing only one scaffold.

The conjugates increase mitochondrial stability against MPT pore opening, with activity equal to or somewhat higher than Dimebon and significantly higher than memantine. In this assay, compounds containing ethylene spacer **IIa** (Fig. **6**) decrease the maximum rate of mitochondrial swelling more efficiently.

Most compounds also exhibit the ability to inhibit acetyl- and butyryl-cholinesterases, as well as potentiate tubulin polymerization. In the last case, the most active were the compounds that contain a fluorine substituent in the R-position for both chemotypes **IIa** and **IIb**, which increase ordered tubulin polymerization by 3-4 times [81].

Thereby, focused tailoring of conjugated structures bearing scaffolds derived from the known neuroactive agents (memantine and dimebon) led to the development of a novel group of multipharmacophore compounds and revealed unexpected properties not inherent to the background agents. The identified lead compounds appear highly promising as multifunctional agents with neuroprotective potential (Table **1**) that simultaneously target several key biological targets involved in the pathogenesis of neurodegenerative processes.

## 4 CONJUGATES OF TETRAHYDRO-ɣ-CARBOLINE DERIVATIVES AND CARBAZOLE/ TETRAHYDROCARBAZOLE

Carbazoles are naturally occurring phytochemicals found in various plants and exhibit a wide range of biological activities. Isolated from the stems and leaves of Clausena lenis, carbazole alkaloids show neuroprotective activities against 6-hydroxydopamine-induced cell death in human neuroblastoma SH-SY5Y cells *in vitro* [85, 86]. Synthetic carbazole derivatives have demonstrated antioxidant activity, the ability to protect neuronal cells against hydrogen peroxide, glutamate, and homocysteic acid-induced cell death, and the capacity to induce neurite outgrowth through the PI3K/Akt signaling pathway [87, 88]. A series of ferulic acid–carbazole conjugates with pronounced antioxidant ability and neuroprotective properties against βA42- and ROS-induced toxicity was designed. Notably, the neuroprotective effect of conjugates was superior to that of the mixture of ferulic acid and carbazole [89]. Neuroprotective activity was also shown for the lead compounds among the multitarget conjugates of carbazole and dopamine D2/D3 receptor agonists, particularly pramipexole. In a cell-based model of Parkinson's disease, these compounds reduce the oxidative stress and cell death induced by neurotoxin 6-hydroxydopamine, inhibit aggregation, and reduce the toxicity of recombinant alpha synuclein protein [90].

Of particular interest is the discovery of the pro-neurogenic effect of aminopropyl carbazole derivatives (so-called P7C3 compounds). It was demonstrated that their pro-neurogenic activity is achieved by protecting newborn neurons from apoptosis by inhibiting mitochondrial permeability transition [91]. P7C3 derivatives also block 1-methyl-4-phenyl-1,-2,-3,-6-tetrahydropyridine (MPTP)-mediated cell death of dopaminergic neurons in Parkinson’s disease [45, 92-94], protect primary neurons from oxygen–glucose deprivation or oxygen–glucose deprivation/reperfusion injury [95], and also possess astrocyte senescence inhibiting properties due to mitophagy increase and suppression of mitoROS [47].

The logical extension of this approach was the attempt to combine a carbazole fragment with another amino-containing (such as aminoadamantane) derivative, or nitrogen-containing heterocycles, such as γ-carboline derivatives, which will be discussed later. Synthesis of this group of conjugates was described in earlier studies [51, 55]. Taking into account the already known targets of such tetrahydro-γ-carboline as Dimebon and it bioisosteric analogues, the effects of such conjugates (series **IIIa** and **IIIb** (Fig. **7**)) on the NMDA-receptors binding sites, activity of acetyl- and butyryl-cholinesterases, functional characteristics of mitochondria, and radical scavenging capacity, as well as the ability of the conjugates to stimulate the assembly of microtubules were studied [55, 96].

It was shown that tetrahydro-γ-carbolines–carbazole conjugates have a low affinity to the intrachannel (MK801-specific) site of the NMDA-receptors; however, unlike Dimebon, they showed higher affinity to the allosteric (ifenprodil-binding) site. Conjugates of the **IIIa** and **IIIb** series do not cause mitochondrial depolarization but reduce mitochondrial swelling. Therefore, these compounds can be assumed to decrease mitochondrial susceptibility to MPT pore opening and to possess neuroprotective potential. As inhibitors of MPT, tetrahydrocarbazole conjugates (**IIIa**) are superior to carbazole conjugates (**IIIb**) [96]. However, even for the lead compounds, the activity of suppressing MPT pore opening is not stronger than that of Dimebon (Table **1**). The lead compound (Fig. **7**) could be positioned as a multi-functional neuroprotective agent: it displays the radical-scavenging activity and the ability to increase mitochondrial resistance to MPT pore induction, but additionally possesses cognitive-stimulating potential as a result of counterbalancing the neurotransmitter deficiency due to BChE inhibition and binding to the NMDA receptor allosteric site [96].

## 5 CONJUGATES OF AMINOADAMANTANE AND CARBAZOLE DERIVATIVES

As a promising option for creating neuroactive polypharmacophore structures, the conjugates of carbazole and aminoadamantane derivatives appear to be a viable option. The synthesis of these new groups of conjugates (Fig. **8**) was recently described [53, 97]. The synthesized compounds were distinguished by substituents in the carbazole ring and different spacers. Two aminoadamantane structures were used as the second component: unsubstituted aminoadamantane (amantadine) and dimethylamino derivative (memantine). The study of conjugates' biological activity includes interactions with the NMDA receptor, evaluation of antioxidant potential (radical-scavenging activity and LP inhibition), determination of effects on mitochondrial membrane potential and calcium-induced swelling as markers of MPT, and on microtubule assembly [98].

Conjugates formed by carbazole derivatives with amantadine do not reveal any affinity to the intrachannel (MK-801-binding) site of NMDA receptor, and unexpectedly, only minor affinity was shown for conjugates with memantine fragment. However, the new conjugates proved to be active towards the ifenprodil-binding site, although the original pharmacophores, carbazoles and adamantanes, do not possess this activity. This activity depends to a greater extent on substituents in the carbazole moiety rather than on the structure of the aminoadamantane moiety. In this case, the combination of the unsubstituted carbazole and amantadine moieties proved to be the most active (IC_50_ = 8.2 µM).

Evaluation of the effect on mitochondrial functions (mitochondrial potential and calcium-induced mitochondrial swelling) did not reveal any significant dependence on substituents in either the carbazole or adamantane moiety. At a concentration of 10 µM, the new conjugates induce a weak mitochondrial depolarization of 10 to 30%. Meanwhile, the study of compounds' action on mitochondrial functions identified several compounds that increase mitochondrial calcium capacity and, at the same time, decrease calcium-induced mitochondrial swelling and depolarization, but without their own influence on mitochondrial potential. On these grounds, these agents could be defined as inhibitors of MPT.

To confirm that the mitochondrial mechanism of action of lead compounds TG-2113x and TG-2112x (Fig. **9**) may underlie their neuroprotective effect, we used a cellular model of non-receptor-mediated calcium overload induced by the calcium ionophore ionomycin. Unexpectedly, virtually complete protection from cell death was observed even at low nanomolar concentrations of these compounds [98].

To study the mitochondrial mechanisms of neuroprotection in a cellular model of excitotoxicity, amantadine conjugate TG2112x, which does not bind to the intracellular site of the NMDA glutamate receptor, was chosen [99]. Glutamate-induced stimulation of calcium uptake in neurons is accompanied by mitochondrial depolarization [100]. However, the presence of TG2112x, even at nanomolar concentrations, eliminates this depolarization almost completely and protects neurons from subsequent death. Further study of the mechanisms of mitochondrial protection revealed that TG2112x exhibits a unique dual effect on mitochondria (Fig. **10**): at low, physiological concentrations of glutamate (5 µM), it can block mitochondrial calcium uptake, thereby increasing the efficiency of the neurotransmitting action of glutamate. On the other hand, at a toxic glutamate concentration (100 µM), compound TG2112x increases the threshold level of calcium concentration required for MPT induction and subsequent neuron death [99]. The same effect was observed for compound TG2113x (Fig. **9**). Additionally, this compound, which contains a memantine fragment, decreases calcium entry into neurons by blocking NMDA receptors. Both agents TG2113х and TG2112х prevent mitochondrial calcium uptake and protect against βA-induced neuronal cell death and mitochondrial depolarization. In *in vivo* experiments, TG2113 protects against age- and scopolamine-induced amnesia in appropriate mouse models [101]. Both compounds had successfully completed preclinical safety trials.

Thus, this class of conjugates was found to possess activity that was not inherent in the starting prototype agents, namely tetrahydrocarbazole and aminoadamantane, specifically binding to the allosteric ifenprodil-binding site of the NMDA receptor and stimulating microtubule assembly formation. The neuroprotective potential of aminoadamantane conjugates and tetrahydrocarbazole derivatives (Table **1**) is caused by a unique dual action on calcium homeostasis and functions in mitochondria [102].

## CONCLUSION

The focused design of novel, effective, multitarget agents for the treatment of neurodegenerative diseases has attracted special attention over the last few decades [33, 34, 72, 103]. It was initially expected that conjugating several neuroactive structural fragments into a single molecule could expand the potential targets involved in disease pathogenesis and provide a more complex therapeutic effect. However, in recent years, it has been revealed that the combination of several pharmacophores in one molecule may not only result in the mere addition of the useful properties of each component, but may also give rise to new types of biological activity, which may promote more effective pharmacological action of such agents [84].

The same patterns are observed about mitochondrial targets, which often determine the neuroprotective potential of compounds (Table **1**). In particular, the conjugates of cycloalkanindoles with phenothiazine (in particular, MB) exhibit mitoprotective properties, namely: an antioxidant effect, prevention of calcium-induced opening of MP pores, and restoration of the electron transport chain of mitochondria, which largely reflect the properties of MB. A similar effect was observed for γ-carboline conjugates with carbazole or aminoadamantane derivatives: their neuroprotective potential-attributable to their ability to inhibit MPT pore opening-is comparable to, or slightly greater than, that of the reference compound Dimebon. However, in the series of original conjugates of tetrahydrocarbazole and aminoadamantane, a novel neuroprotective effect was observed. The compounds TG2112х and TG2113х, chosen as the lead-compound among tetrahydrocarbazole and aminoadamantane conjugates, display a unique feature: the ability to block the mitochondrial calcium uptake at low (physiological) glutamate concentrations and therefore provide neuroprotective effect, to increase the efficiency of neurotransmission, and to potentiate the mitochondrial protective function at high (excitotoxic) glutamate concentrations. Thereby, it can be expected that new medications based on these molecules could provide both neuroprotective and cognition-enhancing properties.

Taken together, it may be concluded that the conjugation of neuroactive pharmacophores into a single molecular structure, in some cases, confers new, promising properties and therefore may be considered a promising approach for creating a novel generation of neuroprotective agents.

## Figures and Tables

**Fig. (1):**
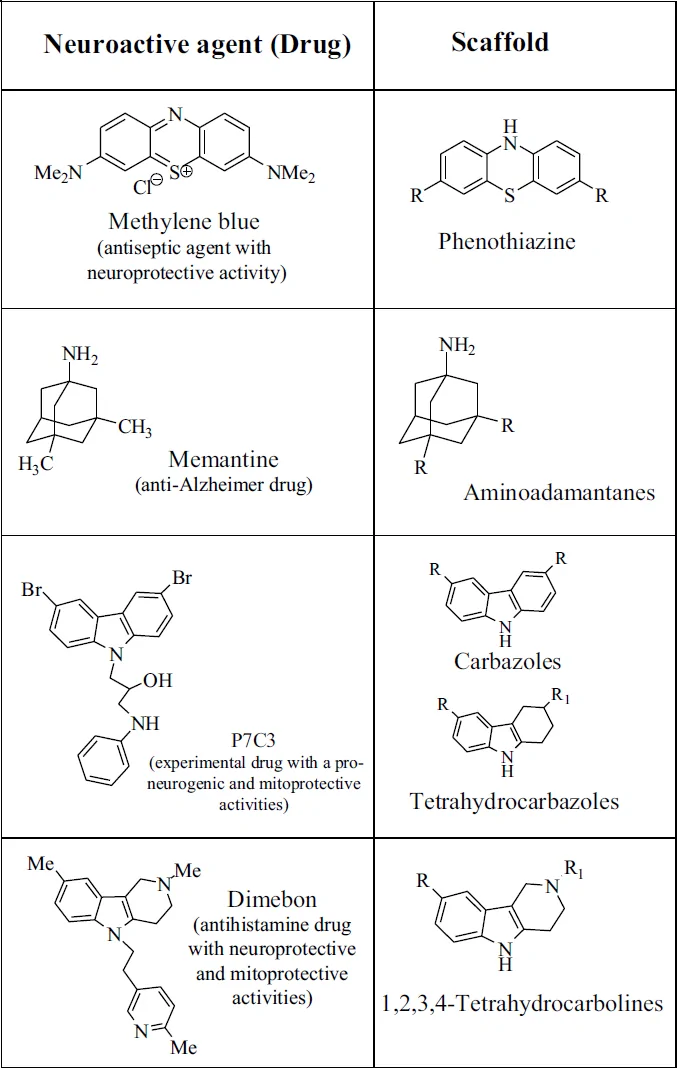
Pharmacophore scaffolds used for the synthesis of conjugated compounds and known drugs that contain these structural moieties.

**Fig. (2):**
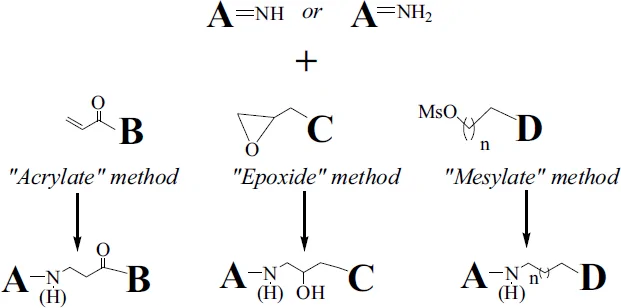
Synthetic algorithms of conjugation of pharmacophore ligands (**A-D**).

**Fig. (3):**
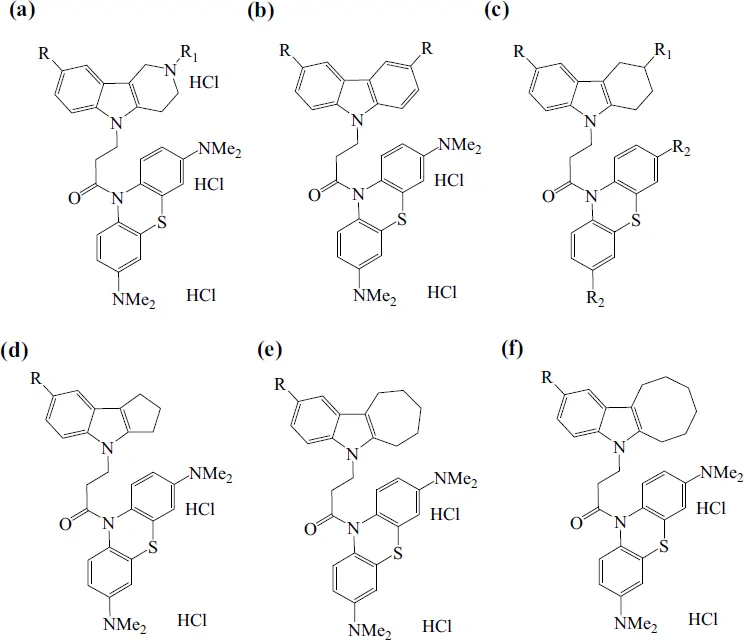
Chemotypes of the newly synthesized conjugates of phenothiazine derivatives with: (**a**) derivatives of tetrahydro-γ-carboline; (**b**) carbazole derivatives; (**c**) tetrahydrocarbazole derivatives; (**d**) 1,2,3,4-tetrahydrocyclopenta[b]indole derivatives; (**e**) 5,6,7,8,9,10-hexahydrocyclohepta[b]indole derivatives and (**f** ) 6,7,8,9,10,11-hexahydro-5H-cycloocta[b]indole derivatives.

**Fig. (4):**
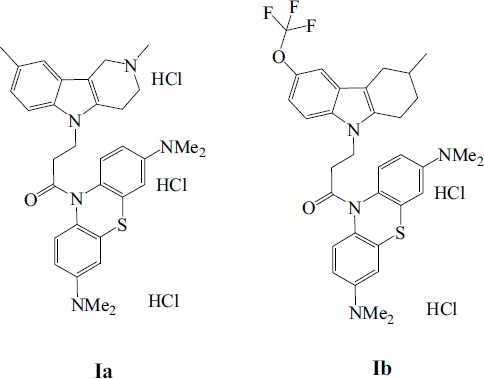
Lead-compounds in a series of MB conjugates with tetrahydrogammacarboline (**Ia**) and carbazole (**Ib**) derivatives that demonstrated the most pronounced antioxidative properties in models *in vitro.*

**Fig. (5):**
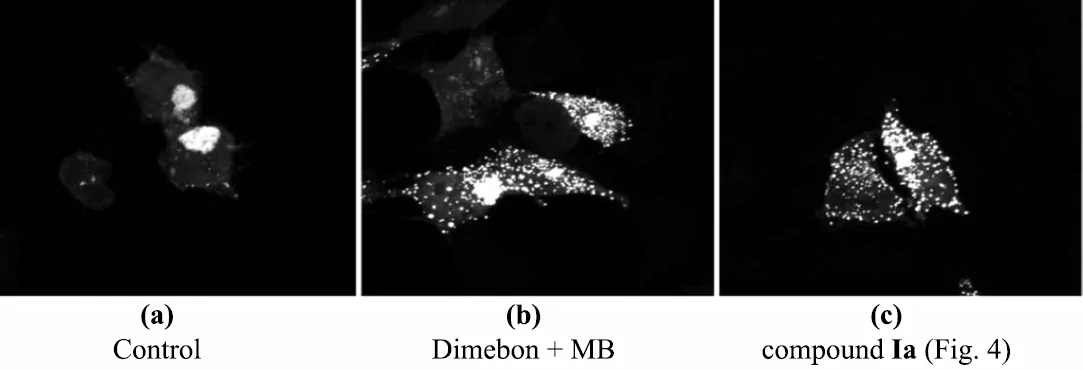
Micrograph of the SH-SY5Y human neuroblastoma cells in the control (**a**), after incubation with a mixture of Dimebon and MB (**b**), and with lead compound Ia (Fig. **4**) (**c**) (all compounds in 1 µM dose) 24 hours after mutFUS-GFP transfection (white spots in the black and white image), with permission from [72].

**Fig. (6):**
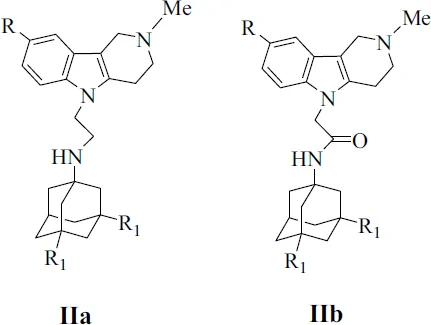
(**IIa** and **IIb**) Structures of conjugates of tetrahydro-ɣ-carboline and aminoadamantane derivatives. R= H, Me, Et; R_1_ = H, Me.

**Fig. (7):**
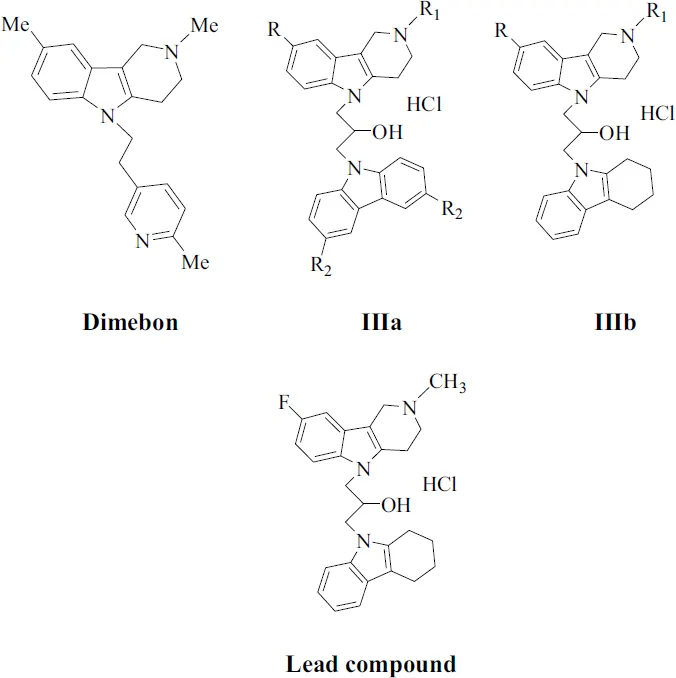
Structures of tetrahydro-ɣ-carboline Dimebon, tetrahydro-ɣ-carboline conjugates with carbazoles (IIIа) and tetrahydrocarbazoles (IIIb).

**Fig. (8):**
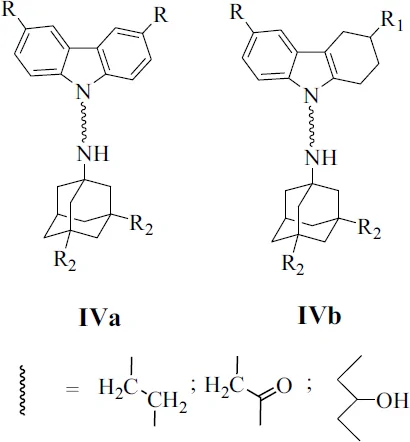
Chemotypes of the newly synthesized conjugates of carbazole and aminoadamantane derivatives.

**Fig. (9):**
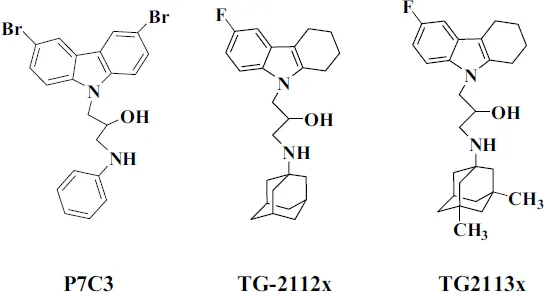
Carbazole prototype with proneurogenic and mitoprotective activity P7C3 [45] and lead-compounds (TG-2112x and TG-2113x) in a series of aminoadamantane conjugates with tetrahydrocarbazole derivatives.

**Fig. (10):**
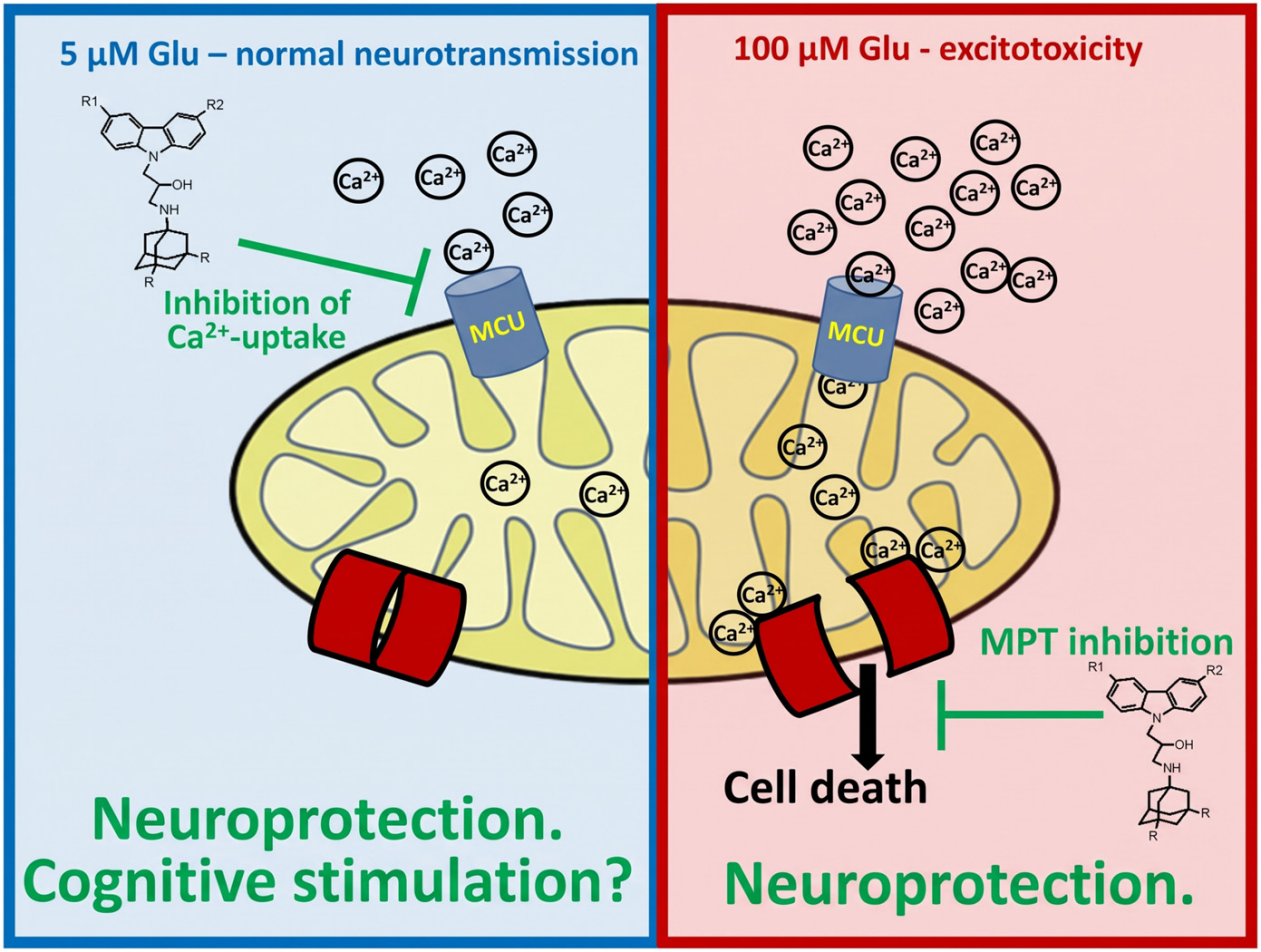
Proposed scheme of dual mitochondrial mechanisms of neuroprotective action of TG2112x and TG2113x. Neuroprotection and stimulation of cognitive functions by lead compounds (TG2112x and TG2113x) may be associated with two main mechanisms of their influence on mitochondria: inhibition of Ca^2+^ uptake and MPT. Under physiological glutamate (GLU) concentrations (approximately 5 µM), the Ca^2+^ levels required to trigger the opening of mitochondrial permeability transition (MPT) pores are not reached; thus, compound interaction with MPT does not occur. Under these conditions, only the inhibition of Ca^2+^ uptake takes place, which may enhance the action of the GLU as neurotransmitter. At toxic GLU concentrations (about 100 µM), the effect of compounds on Ca^2+^ uptake is not sufficient due to cell overloading with Ca^2+^. However, under these conditions, we observe another mechanism of compounds' “mitoprotective” effects: the inhibition of MPT pore opening, accompanied by an increase in mitochondrial calcium capacity.

**Table 1:** Main features of multipharmacophoric conjugates as potential neuroprotectors.

**Compounds**	**Activities**		**References**
**Conjugates of**			
Cycloalkaneindoles (tetrahydro-ɣ-carboline, tetrahydrocarbazole) derivatives and phenothiazine (MB fragment)	Antioxidant activity. Bypass the inhibition of complex I of the mitochondrial respiratory chain. Inhibition of MPT. Protective properties in a cellular model of calcium overload. Inhibition of the FUS protein overexpressed in SH-SY5Y cells aggregation.		[65] [67] [68]
Tetrahydro-ɣ-carboline and aminoadamantane derivatives	Affinity to the MK-801-binding site of the NMDA-receptor, similar to aminoadamantane pharmacophore. Binding to the ifenprodil site of the NMDA receptor, unlike both pharmacophores. Inhibition of MPT.		[84]
Tetrahydro-ɣ-carboline and carbazole/tetrahydrocarbazole derivatives	Low affinity to the intrachannel (MK801-specific) site of the NMDA-receptors. High affinity to the ifenprodil-binding site of the NMDA-receptors. Suppression of MPT.		[96]
Carbazole/ tetrahydrocarbazole derivatives and aminoadamantane derivatives	No (amantadine) or only minor (memantine) affinity to the intrachannel (MK-801-binding) site of NMDA receptor. Binding to the ifenprodil site of the NMDA receptor, unlike both pharmacophores. Lead-compounds TG-2113x and TG-2112x: Neuroprotection in a cellular model of excitotoxicity. Unique dual effect on mitochondria – inhibition of mitochondrial calcium uptake at physiological calcium concentrations and increasing the limit threshold level of calcium concentration needed for MPT. Prevention of glutamate and βA-induced neuronal cell death		[99-101]
Ferulic acid–carbazole derivatives	Antioxidant activity. Neuroprotective against the βA42- and ROS-induced toxicity.		[89]
Pramipexole - carbazole derivatives	Reduction of the oxidative stress and cell death induced by neurotoxin 6-hydroxydopamine. Reduction of aggregation and toxicity of recombinant alpha synuclein protein		[90]
**Pharmacophores used – Derivatives of**			
Tetrahydro-ɣ-carboline	Inhibition of MPT. Antioxidant activity. Anti-NMDA- activity		[49] [73-78]
Bivalent gamma-carbolines conjugates	Inhibiting calcium currents through NR1-1a/NR2A glutamate receptors. Inhibition of MPT. Neuroprotective activity in a cellular model of calcium overload		[79] [80]
Aminoadamantane	Memantine	Inhibiting the intrachannel site of the NMDA-receptor; Inhibition of MPT; Increases the calcium retention capacity of mitochondria; Induce mitophagy and autophagy; Prevent disturbances in mitochondrial dynamics	[16] [81]
	Amantadine	Minor affinity to the intrachannel site of the NMDA-receptor; Only in conditions of impaired mitochondrial function due to HCV-infection restore mitochondrial potential and the calcium threshold required to trigger MPT and revert HCV protein-mediated decrease of mitochondrial oxygen consumption and complex I and V activity; There is the assumption about inhibition of the mitochondrial calcium uniporter (MCU)	[82]
Carbazole	Neuroprotective activities against 6-hydroxydopamine-induced cell death in SH-SY5Y cells. Antioxidant activity. Protection against the hydrogen peroxide, glutamate, and homocysteine-induced cell death. Induction of neurite outgrowth. Aminopropyl carbazole derivatives inhibit MPT and so reveal proneurogenic activity. Block MPTP-, oxygen–glucose deprivation/reperfusion-induced cell death. Inhibit astrocyte senescence due to increasing mitophagy and suppression of mitoROS		[85, 86] [87, 88] [91] [95] [47]

## LIST OF ABBREVIATIONS

AD: Alzheimer’s Disease
ALS: Amyotrophic Lateral Sclerosis
MCU: Mitochondrial Calcium Uniporter
MPT: Mitochondrial Permeability Transition
ND: Neurodegenerative Diseases
PD: Parkinson’s Disease
